# Selective labelling and eradication of antibiotic-tolerant bacterial populations in *Pseudomonas aeruginosa* biofilms

**DOI:** 10.1038/ncomms10750

**Published:** 2016-02-19

**Authors:** Song Lin Chua, Joey Kuok Hoong Yam, Piliang Hao, Sunil S. Adav, May Margarette Salido, Yang Liu, Michael Givskov, Siu Kwan Sze, Tim Tolker-Nielsen, Liang Yang

**Affiliations:** 1 Singapore Centre for Environmental Life Sciences Engineering (SCELSE), Nanyang Technological University, Singapore 637551, Singapore; 2 NUS Graduate School of Integrative Sciences and Engineering, National University of Singapore, Singapore 117543, Singapore; 3 Interdisciplinary Graduate School, Nanyang Technological University, Singapore 637551, Singapore; 4 School of Biological Sciences, Division of Structural Biology and Biochemistry, Nanyang Technological University, Singapore 639798, Singapore; 5 Costerton Biofilm Center, Department of Immunology and Microbiology, University of Copenhagen, 2200 København N, Denmark

## Abstract

Drug resistance and tolerance greatly diminish the therapeutic potential of antibiotics against pathogens. Antibiotic tolerance by bacterial biofilms often leads to persistent infections, but its mechanisms are unclear. Here we use a proteomics approach, pulsed stable isotope labelling with amino acids (pulsed-SILAC), to quantify newly expressed proteins in colistin-tolerant subpopulations of *Pseudomonas aeruginosa* biofilms (colistin is a ‘last*-*resort' antibiotic against multidrug-resistant Gram-negative pathogens). Migration is essential for the formation of colistin-tolerant biofilm subpopulations, with colistin-tolerant cells using type IV pili to migrate onto the top of the colistin-killed biofilm. The colistin-tolerant cells employ quorum sensing (QS) to initiate the formation of new colistin-tolerant subpopulations, highlighting multicellular behaviour in antibiotic tolerance development. The macrolide erythromycin, which has been previously shown to inhibit the motility and QS of *P. aeruginosa*, boosts biofilm eradication by colistin. Our work provides insights on the mechanisms underlying the formation of antibiotic-tolerant populations in bacterial biofilms and indicates research avenues for designing more efficient treatments against biofilm-associated infections.

## Abstract (web summary)

Pathogenic bacteria can aggregate to form biofilms and develop tolerance to antibiotics. Here, the authors use a proteomics approach to study the development of tolerance to the antibiotic colistin in *Pseudomonas aeruginosa* biofilms, and show that co-treatment with a second compound kills the tolerant cells.

## Graphical abstract

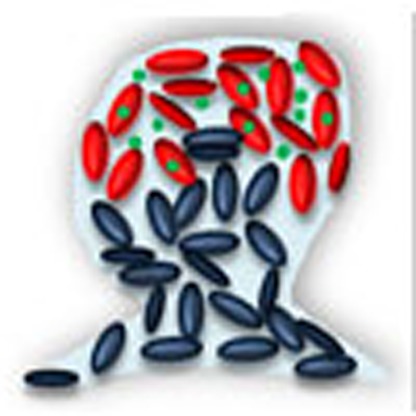

The therapeutic efficacy of antibiotics can be severely crippled by two major bacterial defence strategies. ‘Resistance' occurs when bacteria acquire genetic mutations or mobile genetic elements that enable growth under conditions of constant antibiotic exposure^1^, while ‘tolerance' is mediated by transient phenotypic variations (based on protein expression) that enable bacteria to survive exposures to the highest deliverable doses of antibiotics^2^,^3^.

While the current view is that development of tolerance is intrinsically linked to the biofilm lifestyle, early experiments conducted on bacteria in the planktonic lifestyle revealed that tolerance can develop to a number of stresses including antibiotics^4^,^5^. However, in contrast to the planktonic mode of life, biofilms are sessile, densely populated multicellular communities embedded in biopolymeric matrix components^6^. A major advantage for bacteria that aggregate into biofilms is that a multitude of matrix components and physiological states offers additional levels of protection against immune defences and antibiotics, which cannot be obtained in the planktonic growth mode^7^,^8^,^9^. It is widely accepted that pathogenic bacteria in the biofilm mode significantly contribute to the development of nosocomial infections as they are colonizing hospital settings and chronic infection sites, where they represent a serious threat to public health^6^. Bacterial cells differentiate into several physiologically distinct subpopulations within a biofilm^10^. Antibiotic treatment often eradicates sensitive subpopulations, but leaves small antibiotic-tolerant subpopulations behind, resulting in recurring infections after antibiotic treatment has been stopped^10^. Antibiotic-tolerant bacterial cells are variants of wild-type cells that can revert back to the wild type when antimicrobial treatment has ceased^11^. Their transient nature and low abundance make it difficult to investigate their tolerance-related physiology.

With only a few studies that have investigated planktonic antibiotic-tolerant cells by pre-isolating them using flow cytometry^12^,^13^,^14^,^15^, the mechanisms underlying the formation of antibiotic-tolerant subpopulation in biofilms have remained elusive. Moreover, these pre-isolation-based strategies are destructive and not feasible for studying the antibiotic-tolerant subpopulations in biofilms. This prompted us to develop an alternative, high-resolution ‘-omics' approach to simultaneously characterize the physiologies of sensitive and antibiotic-tolerant subpopulations in biofilms.

*Pseudomonas aeruginosa* forms biofilm with extreme tolerance to antibiotics in nosocomial infections, such as pneumonia and surgical site infections, prompting the Centers for Disease Control and Prevention to classify *P. aeruginosa* under ‘serious'-threat level (http://www.cdc.gov/drugresistance/threat-report-2013/) and the Infectious Diseases Society of America to declare it as one of the six ‘top-priority dangerous, drug-resistant microbes'^16^. Colistin is a last-resort polymyxin antibiotic available for treatment of infections caused by drug-resistant Gram-negative bacteria^17^. *P. aeruginosa* biofilms in flow chambers develop colistin-tolerant cells rapidly and these cells feature high expression levels of the *pmr* operon^18^.

Here we observe the dynamic development of drug-tolerant subpopulations in *P. aeruginosa* biofilms after treatment with colistin. To obtain knowledge about the physiology of the colistin-tolerant biofilm cells, we apply pulsed stable isotope labelling with amino acids (pulsed-SILAC) proteomics method to selectively quantify the newly expressed proteins that incorporate and are thus labelled with heavy C13 lysine, to promote understanding of the colistin-tolerant subpopulation physiology. The colistin-tolerant populations are able to migrate to the top of the dead biofilm by employing type IV pili-dependent motility and initiate formation of new biofilm via quorum sensing (QS)-regulated group activity. Synergistic treatment by use of erythromycin, which can inhibit QS and motility with colistin, is able to boost the elimination of *P. aeruginosa* biofilms. Hence, our study provides key insights in developing novel treatments against biofilm-associated infections, which otherwise prove hard to eradicate.

## Results and Discussion

### Development of colistin-tolerant subpopulations in biofilms

**Figure 1:**
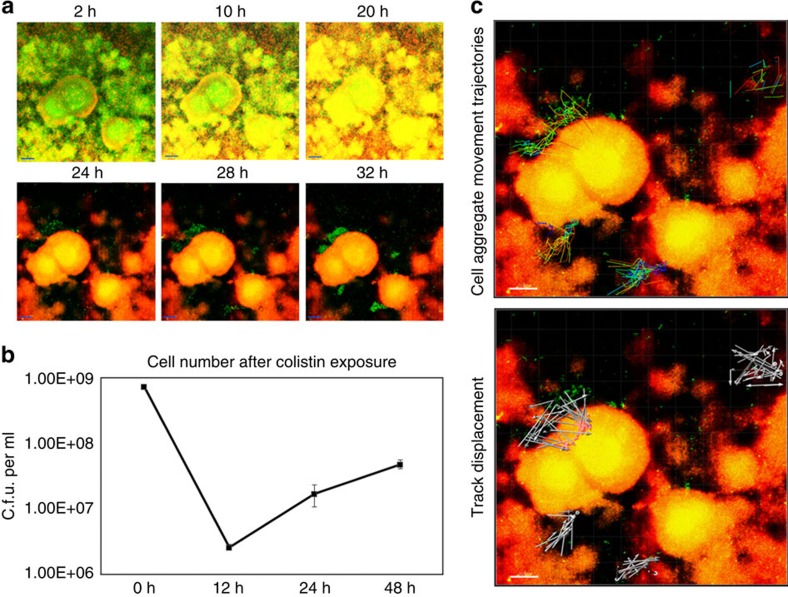
The migration and formation of colistin-tolerant subpopulations in biofilm. (**a**) *P. aeruginosa* PAO1 wild-type biofilms were treated with minimal medium containing 10 μg ml^−1^ colistin followed by real-time CLSM observation from 2 to 32 h of treatment. Scale bars, 50 μm. Colistin-tolerant cells in *P. aeruginosa* PAO1 biofilms migrated onto the dead biofilm and formed a live colistin-tolerant biofilm. Culture medium flow through on top of the biofilms from the top of image. Experiments were performed in triplicate, and a representative image for each condition is shown. Live cells appear green, whereas dead cells appear red or yellow. Videos of the migration and formation of colistin-tolerant biofilm are available in online Supplementary Videos 1 and 2. (**b**) C.f.u. per ml of the PAO1 biofilms after 0, 6, 24 and 48 h of colistin treatment. The means and s.d. from three experiments were shown. (**c**) Movement trajectories and track displacement of live colistin-tolerant cell aggregates moving onto the dead biofilm. Culture medium flow through on top of the biofilms from top of image. Scale bars, 10 μm.

We monitored the development of live and dead subpopulations of *P. aeruginosa* biofilms after exposure to colistin in real-time by using time series confocal image acquisition as well as enumeration of viable cells with colony forming units (c.f.u. per ml). Colistin exposure of *P. aeruginosa* wild-type biofilms tagged with green fluorescent protein (GFP) led to a sudden reduction in cell viability according to the propidium iodide (PI) dead staining (Fig. 1a and Supplementary Movies 1 and 2) and c.f.u. per ml counting (Fig. 1b). However, we noticed that a few *P. aeruginosa* cells localized at the substratum of the biofilms remained alive even after 24 h of colistin treatment (Fig. 1a), which might result from the heterogeneous compositions/structures of the exopolymeric matrix materials^19^,^20^. Certain exopolymeric matrix components might act as physical barriers and reduce the penetration of colistin into deep part of biofilms^21^, which allows a few biofilm cells at the substratum to survive and acquire colistin tolerance.

Interestingly, we observed that colistin-tolerant subpopulations were able to migrate towards the top of the large microcolonies of dead *P. aeruginosa* biofilm cells (Fig. 1a,b and Supplementary Movies 3 and 4). Tracking of colistin-tolerant cell aggregates close to the large microcolonies revealed that those aggregates moved towards the dead microcolonies (Fig. 1c and Supplementary Movies 3 and 4)—a migration that was unrelated to the flow direction of the culture medium (from top to bottom of the image). In contrast, single colistin-tolerant cells far away from the large microcolonies appeared randomly distributed (Fig. 1c and Supplementary Movies 3 and 4). These results suggested that migration of colistin-tolerant cell aggregates towards large microcolonies of dead biofilm cells was a coordinated process that might involve specific signals.

Colistin-tolerant cells obtained from biofilms only showed transient expression of the *pmr* operon (Supplementary Fig. 1a) and lost their colistin tolerance after overnight culturing in ABTGC medium containing no colistin (Supplementary Fig. 1b,c), supporting our view that the colistin-tolerant cells were the result of phenotypic variation and not *per se* resistant cells. DNA sequencing of the biofilms with and without colistin treatment showed that there was no convergent non-synonymous mutation gained by the three colistin-treated biofilm populations compared with the control biofilm populations (Supplementary Data 1). The development of motile colistin-tolerant subpopulations in biofilms has major clinical implications as they can result in persistent infections.

### Using pulsed-SILAC to study colistin-tolerant subpopulations

**Figure 2:**
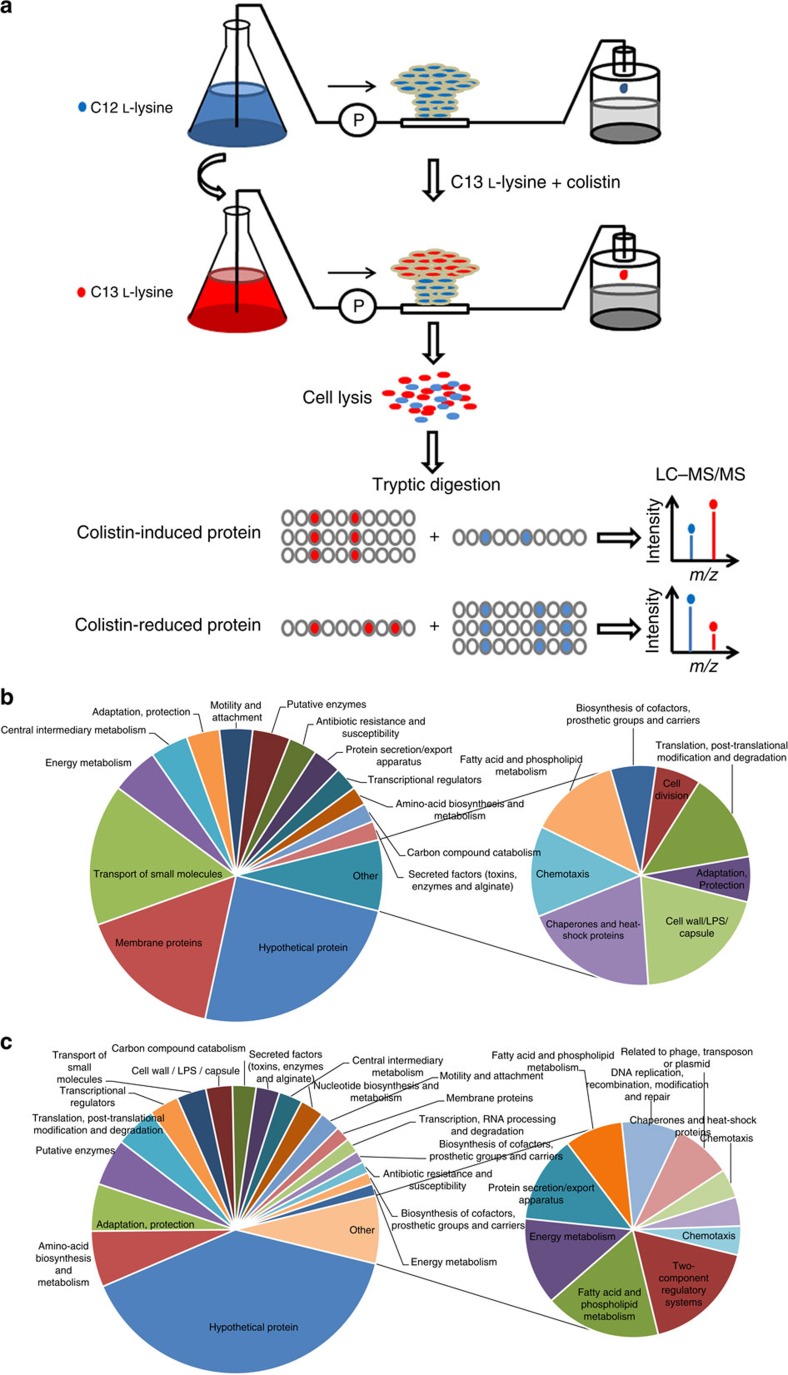
Workflow of pulsed-SILAC proteome analysis of antibiotic-tolerant and -sensitive subpopulations. (**a**) Biofilms were grown in medium containing C12 L-lysine for 72 h and were then treated with medium containing 10 μg ml^−1^ colistin for 6 h to allow the development of colistin-tolerant cells. Colistin-tolerant subpopulations were then treated with medium containing C13 L-lysine and 10 μg ml^−1^ colistin to label the tolerant cells with C13. Cells were collected, and pulsed-SILAC proteome analysis was conducted to determine the protein abundance in the colistin-tolerant cells in which the new synthesized proteins are tagged with C13 L-lysine, while the proteome of colistin-susceptible biofilm cells contain the normal C12 L-lysine. Downregulated: lowly expressed proteins in the colistin-tolerant cells (**b**); and upregulated: highly expressed proteins in the colistin-tolerant cells (**c**); proteins in antibiotic-tolerant cells were classified into function groups.

To further understand the process that caused the directed motility and formation of colistin-tolerant biofilm subpopulations, we further used the pulsed-SILAC quantitative proteomic approach, with the aim of studying new proteins' abundance in the colistin-tolerant subpopulations formed by *P. aeruginosa* biofilms after a high-dose colistin treatment (Fig. 2a). We hypothesized that the actively expressed proteins in the colistin-tolerant subpopulations provided the survival advantages to the bacteria. SILAC is a simple and fast but powerful *in vivo* method, commonly used for eukaryotes to label proteins for mass spectrometry (MS)-based quantitative proteomics^22^. Although this method had been used in labelling prokaryotes to compare biofilm and planktonic cells^23^,^24^, pulsed-SILAC had never been employed to determine new proteins' abundance in the antibiotic-sensitive and -tolerant subpopulations coexisting in the same bacterial biofilm.

A *P*. *aeruginosa* mutant that cannot synthesize lysine, mPAO1Δ*lysA*, was employed to form biofilms in medium supplemented with C12 L-lysine. Although this mPAO1Δ*lysA* mutant could only grow in the presence of L-lysine (Supplementary Fig. 2), it formed biofilms similar to wild-type mPAO1 when lysine was added to the culture medium (Supplementary Fig. 3). Biofilms were treated with high doses of colistin at 10 μg ml^−1^ (10-fold higher than the minimum bactericidal concentration) in minimal medium supplemented with C12 L-lysine for 8 h. After killing the antibiotic-sensitive population, the remaining surviving antibiotic-tolerant cells were then treated with fresh medium containing colistin and C13 L-lysine for 48 h to incorporate C13 L-lysine into the newly expressed proteins of only live cells (Fig. 2a).

This pulsed-labelling approach allowed for selective tagging of newly expressed proteins in the antibiotic-tolerant subpopulations, with C13 L-lysine without prior physical isolation. The level of new protein abundance could be normalized by dividing the relative abundances of C13 L-lysine-tagged proteins (proteins expressed after colistin treatment) with the relative abundances of C12 L-lysine-tagged proteins (proteins expressed before colistin treatment).

We used Q Exactive MS to analyse the pulsed-SILAC labelled samples, followed by the MaxQuant and Andromeda software^25^,^26^ to identify 4,250 *P. aeruginosa* proteins with <1% false discovery rate (FDR). Only proteins that were significantly and consistently changed/regulated in three biological replicates (variability <30%) were shortlisted for further analysis (Supplementary Data 2). Lowly and highly expressed proteins in the colistin-tolerant subpopulation as compared with the colistin-sensitive population are functionally grouped in Fig. 2b,c, respectively, while the complete list of lowly and highly expressed proteins are listed in Supplementary Data 3 and 4, respectively.

Our initial analysis of the data showed that some of the actively expressed proteins were subunits of ribosomes (Supplementary Data 4), supporting the notion that colistin-tolerant cells are metabolically active^27^. We also found that the bifunctional polymyxin-resistance proteins ArnA and PmrA were expressed at a high level in antibiotic-tolerant cells (Supplementary Data 2, markers intensity sheet: ArnA (intensity L:H=1.51E+10:4.58E+10); PmrA (intensity L:H=5.26E+09:1.12E+10)). The *arnBCADTEF* lipopolysaccharide-modification operon had been shown to be controlled by the upstream *pmr* operon and contribute to the tolerance to polymyxin B and colistin in *P. aeruginosa*^28^. Hence, both proteins served as positive controls and showed that pulsed-SILAC was highly reliable in detecting proteins important in colistin tolerance. These two markers were consistently detected with high abundance level in the heavy C13 lysine-labelled proteins in seven out of eight technical replicates of the three biological replicates except in BR1:R2, where ArnA was not detected in the light C12 condition, and PmrA was not detected in both H and L conditions due to technical variation (Supplementary Data 2, markers intensity sheet). The missing detection of marker proteins in BR1:R2 might be attributed to growth variations during biofilm cultivation as biofilm experiments normally require a long cultivation period (5 days).

### Pili and QS led to colistin tolerance

**Figure 3:**
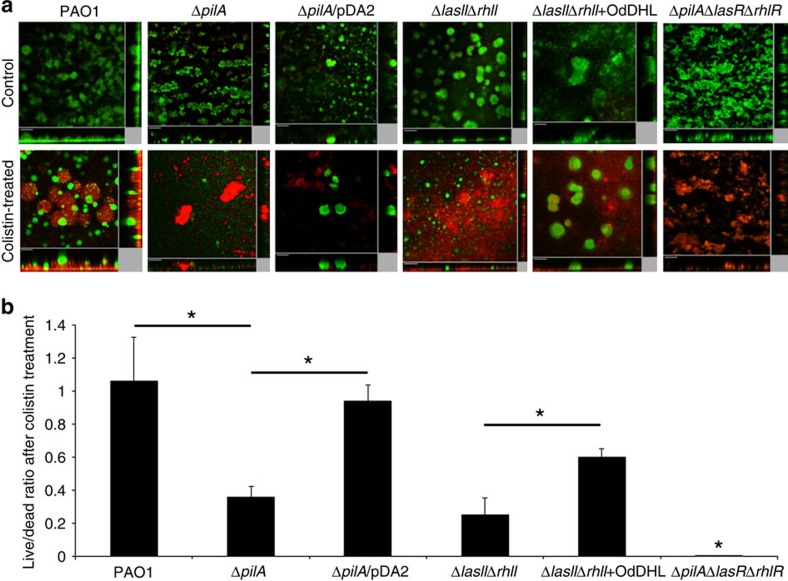
The role of pili and QS in the development of antibiotic-tolerant subpopulations in biofilms. (**a**) Biofilms were cultivated for 72 h using *P. aeruginosa* PAO1, Δ*pilA*, Δ*pilA*/pDA2, Δ*lasI*Δ*rhlI*, Δ*lasI*Δ*rhlI*+OdDHL and Δ*pilA*Δ*lasR*Δ*rhlR* strains, followed by treatment with medium containing 10 μg ml^−1^ colistin. No migration of tolerant subpopulation was observed for Δ*pilA* mutant biofilms, and the majority of the cells in Δ*pilA* biofilms were killed by colistin. Complementation of Δ*pilA* with pDA2 restored the antibiotic-tolerant subpopuation development after colistin treatment. The QS-defective Δ*lasI*Δ*rhlI* mutant biofilms could only develop small amount of antibiotic-tolerant subpopulation after colistin treatment. Addition of OdDHL partially restored the development of antibiotic-tolerant subpopulation after colistin treatment. The pili and QS-defective Δ*pilA*Δ*lasR*Δ*rhlR* mutant biofilms were unable to develop antibiotic-tolerant subpopulation after colistin treatment. The central images show horizontal optical sections, whereas the flanking images show vertical optical sections. Live cells appear green and dead cells appear red. Scale bars, 50 μm. (**b**) Live/dead ratios of 72-h biofilms formed by *P. aeruginosa* PAO1, Δ*pilA*, Δ*pilA*/pDA2, Δ*lasI*Δ*rhlI*, Δ*lasI*Δ*rhlI*+OdDHL and Δ*pilA*Δ*lasR*Δ*rhlR* strains after colistin treatment. The mean and s.d. from three experiments is shown. *P<0.01, Student's *t*-test.

Interestingly, we found that antibiotic-tolerant subpopulations also expressed proteins required for type IV pili assembly, such as PilF (ref. 29, at high levels (Supplementary Data 4). Hence, we hypothesized that type IV pili are required for the migration of colistin-tolerant cell aggregates onto the dead microcolonies. Migration of colistin-tolerant subpopulations was decreased in biofilms formed by the type IV pili mutant, Δ*pilA* (Fig. 3a,b). Complementation of Δ*pilA* with pDA2, a plasmid containing the *pilA* gene in a pUCP18 vector, restored the antibiotic-tolerant phenotype (Fig. 3a,b).

Furthermore, we found that QS-regulated proteins, including LasB, chitinase and phenazine/pyocyanin-synthesis (Phz) proteins, were highly expressed in colistin-tolerant antibiotic-tolerant subpopulations (Supplementary Data 4). Because QS coordinates group behaviour in biofilms^30^, we thus hypothesized that QS coordinates the development of antibiotic-tolerant microcolonies within biofilms. The QS null mutant, Δ*lasI*Δ*rhlI*, developed less antibiotic-tolerant subpopulations in response to colistin treatment than the wild type (Fig. 3a,b). Chemical complementation by the addition of 1 μM N-3-(oxododecanoyl)-L-homoserine lactone (OdDHL) (a QS signalling molecule) to Δ*lasI*Δ*rhlI* biofilms allowed Δ*lasI*Δ*rhlI* to regain the development of colistin-tolerant subpopulations (Fig. 3a,b). We further showed that biofilms formed by a QS and type IV pili-defective triple mutant, Δ*pilA*Δ*lasR*Δ*rhlR*, could only develop a negligible amount of antibiotic-tolerant subpopulations in response to colistin treatment as compared with the wild type (Fig. 3a,b).

**Figure 4:**
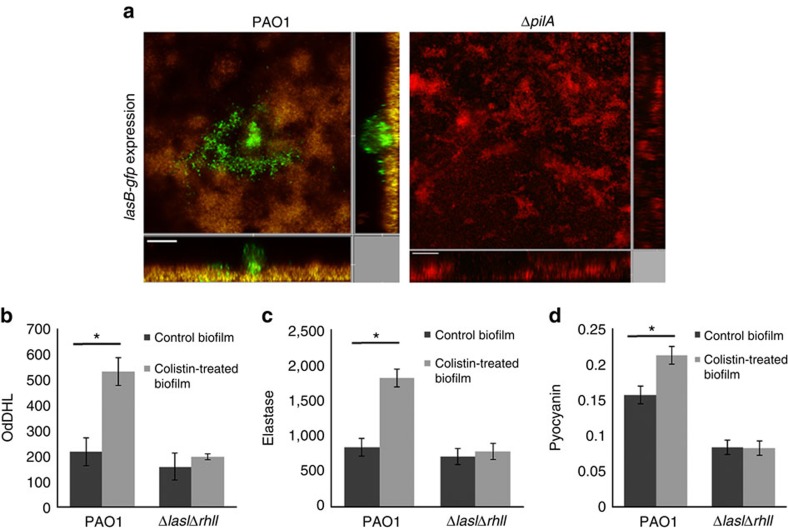
QS-related products are upregulated in antibiotic-tolerant subpopulations in *P. aeruginosa* biofilms. (**a**) The 72-h biofilms formed by the *P. aeruginosa* PAO1 and Δ*pilA* containing the *lasB-gfp* translational fusion were treated with medium containing 10 μg ml^−1^ colistin for 24 h followed by CLSM observation. The *lasB-gfp* translational fusion was induced to high levels in PAO1 colistin-tolerant cells but was not observed in Δ*pilA* biofilms. Experiments were performed in triplicate, and a representative image for each condition is shown. Live cells appear green, whereas dead cells appear yellow or red. The central images show horizontal optical sections, whereas the flanking images show vertical optical sections. Scale bars, 50 μm. (**b**) Antibiotic-tolerant cells from PAO1 biofilms secreted more OdDHL than untreated biofilm cells. (**c**) Antibiotic-tolerant cells from PAO1 biofilms produced more elastase than untreated biofilm cells. (**d**) Antibiotic-tolerant cells from PAO1 biofilms produced more pyocyanin than untreated biofilm cells. The mean and s.d. from three experiments is shown. *P<0.01, Student's *t*-test.

We found that type IV pili-mediated migration preceded induction of QS activity in the colistin-tolerant subpopulations. The QS reporter *lasB-gfp* translational fusion^31^ was highly expressed in antibiotic-tolerant subpopulations of wild-type biofilms but not in the antibiotic-tolerant subpopulations of Δ*pilA* biofilms in response to colistin exposure (Fig. 4a). Colistin-tolerant biofilms produced more OdDHL, the QS autoinducer, than wild-type biofilms (Fig. 4b). In response to colistin treatment, colistin-tolerant wild-type biofilms also secreted more elastase and pyocyanin than untreated biofilms, which correlates with our proteomic data that LasB and pyocyanin-synthesis proteins were highly expressed in colistin-tolerant biofilm cells (Fig. 4c,d).

Given the active expression of virulence factors in the colistin-tolerant subpopulations, we evaluated the macrophage-killing capacity of supernatants from wild-type biofilms with and without colistin treatment by using the mouse RAW264.7 cell line. After staining the dead macrophages with 20 μM PI, we observed that the supernatant from colistin-treated biofilms was more cytotoxic to macrophages than that of control biofilms (Supplementary Fig. 4). Induction of QS-regulated virulence factors in colistin-tolerant microcolonies is clinically relevant because they are detrimental to the host immune system^32^,^33^,^34^,^35^,^36^.

It is unclear why the colistin-tolerant cell aggregates migrate to the top of dead biofilms. Our previous work demonstrated that the motile subpopulation of the wild-type *P. aeruginosa* biofilms migrate towards the non-motile subpopulation to seek iron source^37^. We hypothesized that the tolerant cells migrate to acquire iron. To test this hypothesis, we added the iron chelator 2,2-dipyridyl (DIPY)^38^ together with colistin to treat *P. aeruginosa* wild-type biofilms. We found that DIPY was able to interfere with the development of colistin-tolerant subpopulations associated with the dead microcolonies (Supplementary Fig. 5).

Hence, in addition to mechanisms directly involved in antibiotic tolerance, we showed that multicellular behaviours such as migration and cell–cell signalling (QS) are important in the recovery of pathogenic biofilms that can survive the otherwise lethal antibiotic treatments. Since clinical departments only test for resistance (growth versus no growth on medium supplemented with antibiotics) before the commencement of antibiotic treatments of the patient, the development of antibiotic-tolerant subpopulations in response to exposures can be considered a concealed mechanism of antibiotic recalcitrance.

### Chemical Approach to combat colistin-tolerant subpopulations

Given that both type IV pili-mediated migration and QS are required for establishing antibiotic-tolerant subpopulations in developing *P. aeruginosa* biofilm, we employed a chemical biology approach to disable these activities. Macrolides, which can inhibit type IV pili assembly and QS in *P. aeruginosa*^39^, were used in combination with colistin to treat *P. aeruginosa* biofilms.

**Figure 5:**
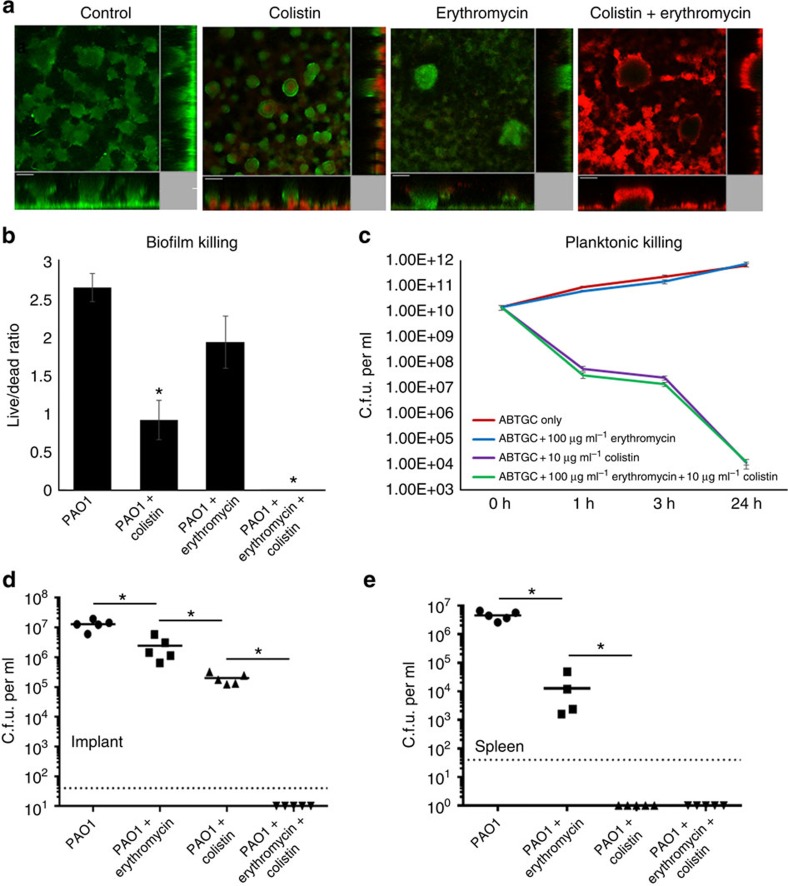
Targeting type IV pili and QS simultaneously leads to eradication of antibiotic-tolerant cells. (**a**) Colistin-tolerant cells formed in PAO1 flow cell biofilms after colistin and erythromycin single treatment and combined treatment. Colistin-tolerant cells were unable to form in PAO1 biofilms after combined erythromycin+colistin treatment. Experiments were performed in triplicate. Live cells appear green and dead cells appear red. Scale bars, 50 μm. (**b**) Live/dead ratios were calculated based on CLSM images. The mean and s.d. from five experiments is shown for *in vivo* biofilms. *P<0.01, One-way ANOVA. (**c**) C.f.u. per ml of PAO1 planktonic cultures treated with colistin, erythromycin and colistin+erythromycin. The mean and s.d. from three experiments is shown. C.f.u. per ml of *in vivo* PAO1 biofilms obtained from implant (**d**) and cells from the spleen (**e**), with and without antibiotic treatment. Dotted horizontal lines represent limit of detection. The mean and s.d. from five experiments is shown for *in vivo* biofilms. *P<0.01, Student's *t*-test.

Treatment with erythromycin had a mild killing effect on biofilm cells and planktonic cells (Fig. 5a–c, respectively), implying that erythromycin was unable to kill the biofilms. However, combination treatments with erythromycin and colistin lead to complete eradication and repression of the development of antibiotic-tolerant subpopulations (Fig. 5a,b). Supporting that our combination treatment was specific to the colistin-tolerant cells present in the biofilm mode of life, we did not observe the synergistic effect of colistin and erythromycin against planktonic *P. aeruginosa* at concentrations equivalent to that of the biofilm treatment (Fig. 5c).

To verify the functional eradication of antibiotic-tolerant cells by both antibiotics *in vivo*, we used a murine model for implant-associated infection^40^. As opportunistic infections on the host could occur when biofilm is formed on implants such as catheters and heart valves, treatments that prevent the rise of antimicrobial-tolerant or -resistant populations are essential^41^,^42^. Implants coated with *P. aeruginosa* biofilms were surgically inserted into the peritoneum of mice. The mice were treated locally with antibiotics to emulate the treatment given to patients with infections from implants. The concentration of antibiotics (1 mg kg^−1^ colistin and 10 mg kg^−1^ erythromycin) used for each mouse was well below the median lethal dose of each antibiotic (86 mg kg^−1^ colistin and 280 mg kg^−1^ erythromycin), ensuring the non-lethal treatment of *P. aeruginosa* biofilm in the mouse.

The c.f.u. per ml count of *P. aeruginosa* from the implant and spleen after 24 h incubation in the mouse revealed that administration of colistin or erythromycin alone did not eradicate the infection. However, synergistic treatments with colistin and erythromycin eradicated the biofilm to the limit of detection by c.f.u. per ml (Fig. 5d). Since we were unable to detect live bacterial cells in the spleen samples, we concluded that the spread of infection was also halted (Fig. 5e). Hence, supplementing conventional antibiotic treatment with a tolerance-interfering compound appears to be a promising therapy for eradicating biofilm-associated infections.

The association between type IV pili and the colistin-tolerant subpopulations in developing *P. aeruginosa* biofilm has been presented previously^18^, whereas the position in a nutrient-rich microenvironment seems to be important for the development of colistin-tolerant subpopulations in more mature *P. aeruginosa* biofilms^27^. We here tracked the formation of antibiotic-tolerant subpopulations in developing *P. aeruginosa* biofilms and used pulsed-SILAC to selectively label their proteome to determine new protein abundance in the antibiotic-tolerant subpopulations, which led to the identification of multiple genes/proteins essential for the development of antibiotic-tolerant biofilm.

**Figure 6:**
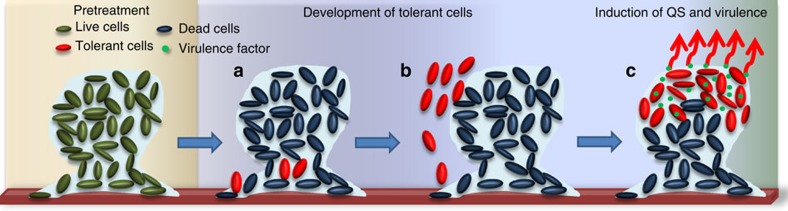
Model of antibiotic-tolerant cell formation in *P. aeruginosa* biofilms. (**a**) Antibiotics such as colistin treatment kill most of the cells in the biofilms, but leave a few antibiotic-tolerant cells at the bottom of the biofilm. (**b**) Antibiotic-tolerant cells expand in numbers and migrate to the top of the biofilm using pilus-mediated motility. (**c**) Assemblies of antibiotic-tolerant cells induce QS that leads to the production of QS-related virulence factors, such as elastase and pyocyanin. A new antibiotic-tolerant biofilm is formed.

We propose the following model for the development of colistin-tolerant subpopulations in developing *P. aeruginosa* biofilm, which links it to the ‘phoenix effect' (Fig. 6): (a) the majority of the biofilm is killed by colistin treatment; (b) surviving antibiotic-tolerant cell aggregates overexpress type IV pili for targeted migration to the top of the dead biofilm; (c) and produce QS-regulated factors for promoting the formation of new antibiotic-tolerant microcolonies. The microcolony size in our *in vitro P. aeruginosa* biofilms is around 50 μm, which is within the range of microcolony sizes identified from *in vivo*^43^,^44^ and *ex vivo*^45^,^46^ *P. aeruginosa* biofilms. This suggested that colistin might have similar effects on *in vivo P. aeruginosa* biofilms as those observed on our *in vitro P. aeruginosa* biofilms.

Furthermore, our study showed that targeting mechanisms important to antibiotic-tolerant cells greatly improved conventional biofilm treatment strategies. It highlighted the importance of developing QS and motility inhibitors that can be given to chronically infected patients, with the aim of constituting functional anti-biofilm chemotherapies. This strategy might also be applied to other complex differentiated communities, such as cancer, as a recent study revealed the presence of motile drug-tolerant cells in chemotherapy-treated cancer cell populations^47^.

## Methods

### Bacterial strains and growth conditions

The bacterial strains and plasmids used in this study are listed in Supplementary Table 1. *P. aeruginosa* strains were grown at 37 °C in ABT minimal medium^48^ supplemented with 5 g l^–1^ glucose (ABTG) or 2 g l^−1^ glucose and 2 g l^–1^ casamino acids (ABTGC). For marker selection in *P. aeruginosa*, 30 μg ml^−1^ gentamicin and 200 μg ml^−1^ carbenicillin were used as appropriate.

### Cultivation of biofilms in flow chambers

MiniTn7-*gfp*-tagged *P. aeruginosa* biofilms were cultivated in ABTG medium at 37 °C using 40 mm × 4 mm × 1 mm three-channel flow chambers. Flow chambers were assembled as described previously, consisting of a flow cell that acted as a chamber for the growth of biofilms^49^. The flow cell was supplied with a flow of medium and oxygen, while waste medium was removed into a waste flask, by using a peristaltic pump. Each flow channel was inoculated with 300 μl of a 1:1,000 dilution of an overnight culture using a syringe and was incubated without flow for 1 h. Medium flow was started and maintained at a velocity of 0.2 mm s^−1^ by the A Cole-Palmer peristaltic pump. After 72 h of growth, PAO1 biofilms were treated with 10 μg ml^−1^ colistin. After 48 h of treatment, 300 μl of 20 μM PI was injected into each flow channel to stain dead cells in the biofilm. Experiments were performed in triplicate, and results are shown as the mean±s.d.

### Reversion of antibiotic-tolerant phenotype in biofilms

MiniTn7-*gfp*-tagged and *pmr*-*gfp*-tagged *P. aeruginosa* biofilms were cultivated and treated with colistin as described above. Colistin-containing ABTG medium was switched to antibiotic-free ABTG to allow the reversion of antibiotic-tolerant phenotype back to normal biofilm for 48 h. To examine whether the antibiotic-tolerant phenotype can be re-induced, the biofilms were challenged with 10 μg ml^−1^ colistin for the second cycle. A volume of 300 μl of 20 μM PI was injected into each flow channel to stain dead cells in the biofilm. Biofilms were observed at 0 and 4 h after the second challenge of colistin.

### Reversion of antibiotic-tolerant cells in planktonic cultures

PAO1 and PAO1/*pmr-gfp* biofilm containing the live colistin-tolerant subpopulations and dead colistin-sensitive subpopulations were obtained from the flow chamber biofilm by flushing out the entire biofilm with 5 ml of 0.9% NaCl. The biofilm was homogenized by vortexing for 30 s and the cells were washed twice with 1 ml 0.9% NaCl. The antibiotic-tolerant cells were grown in 2 ml ABTGC to revert to normal phenotype and 2 ml ABTGC with 10 μg ml^−1^ colistin to maintain the colistin-tolerance phenotype, at 37 °C, 200 r.p.m. for 16 h. The cells were then washed twice in 2 ml 0.9% NaCl. PAO1 cells were serially diluted, plated on Luria–Bertani (LB) agar and incubated at 37 °C overnight. C.f.u. per ml was calculated by multiplying the average number of colonies by the dilution factor and dividing by the volume. For PAO1/*pmr-gfp* cells, fluorescence from *pmr-gfp* expression (expressed in relative fluorescence units, r.f.u.) was measured for each well using a microplate reader (Tecan Infinite 2000) and was normalized to the OD_600_ of each well. Experiments were performed with three replicates, and the results are shown as the mean±s.d.

### Viable colony counts of biofilms with colistin treatment

PAO1 biofilms were cultivated as described above. After 72 h of growth, PAO1 biofilms were treated with 10 μg ml^−1^ colistin. Biofilms were collected from flow chamber by mechanical disruption with syringes at 0, 6, 24 and 48 h of colistin treatment. The biofilms were resuspended in 0.9% NaCl and further homogenized by vortexing. PAO1 cells were serially diluted, plated on LB agar and incubated at 37 °C overnight. C.f.u. per ml was calculated by multiplying the average number of colonies by the dilution factor and dividing by the volume. Experiments were performed with three replicates, and the results are shown as the mean±s.d.

### Microscopy and image acquisition of biofilms

All microscopy images were captured and acquired using an LSM confocal laser scanning microscope (CLSM; Carl Zeiss, Germany). The × 20 objective was used to monitor GFP and PI fluorescence. IMARIS software (Bitplane AG, Zurich, Switzerland) was used to process the images. Experiments were performed in triplicate, and representative images are shown.

### Video of antibiotic-tolerant cell migration in biofilms

MiniTn7-*gfp*-tagged PAO1 biofilms were treated with 10 μg ml^−1^ colistin and 4 μg ml^−1^ PI. From 24 to 32 h of colistin treatment, videos were captured and acquire using a CLSM with a × 40 objective lens. GFP and PI fluorescence were observed. IMARIS software (Bitplane AG, Zurich, Switzerland) was used to process the videos. Experiments were performed in triplicate, and representative videos are shown.

### Tracking antibiotic-tolerant cells migration in biofilms

After acquiring videos as described in previous section, IMARIS software (Bitplane AG) was used to process the particle tracking of antibiotic-tolerant cells migrating, according to the manufacturer's instructions.

### DNA sequencing analysis

Genomic DNA of the *P. aeruginosa* biofilms with and without 48 h colistin treatment was purified using QIAamp DNA Mini Kit (Qiagen, Germany) and sequenced on an Illumina MiSeq V3 platform generating 300 bp long paired-end reads. The experiment was performed in three biological replicates: three colistin-treated biofilms and three control biofilms. The average insert sizes are 490–544 nucleotides and the average genomic coverage depths are 63–167 folds. Nucleotide differences were generated from the CLC Genomics Workbench 8.0 (CLC Bio, Aarhus, Denmark), and all of the used parameters were listed in Supplementary Methods. Briefly, adapters and low-quality reads were trimmed off. Paired-end reads in FASTQ format of colistin-treated and control biofilm genomes were first mapped against the *P. aeruginosa* PAO1 genome (NC_002516). Variants were detected using the low-frequency variant detection method with the required significance of 1%. Convergent non-synonymous variants of colistin-treated biofilm genomes compared with the *P. aeruginosa* PAO1 reference genome were obtained with the following criterions: (i) Parallel variants were found in the same gene in all of the three colistin-treated biofilm samples; and (ii) no variant was found in the same gene in any of the control biofilm samples.

### Pulsed-SILAC

Three independent biological replicates (BR1, BR2 and BR3) were performed. Biofilms treated with colistin were grown and prepared for pulsed-SILAC analysis^22^. PAO1 Δ*lysA* biofilms were cultivated in 25 cm × 5 mm Ø flow tubes as described previously^49^, using ABTG medium, 500 μM C12 L-lysine and 192 mg l^−1^ Amino acid Drop-out Mix Minus Lysine without Yeast Nitrogen Base (United States Biological, MA) at 37 °C. Flow channels were inoculated with 1 ml of a 1:1,000 dilution of an overnight culture using a syringe and were incubated without flow for 1 h. A Cole–Palmer peristaltic pump was used to supply medium at a velocity of 0.2 mm s^−1^, which corresponds to a laminar flow with a Reynolds number of 0.02. After culturing for 72 h, biofilms were treated with 10 μg ml^−1^ colistin for 6 h to kill the antibiotic-sensitive cells in biofilms. Biofilms were then treated with fresh ABTG supplemented with 500 μM C13 L-lysine and 192 mg l^−1^ Amino acid Drop-out Mix minus Lysine without Yeast Nitrogen Base for 48 h to tag new synthesized proteins in live antibiotic-tolerant cells with C13 L-lysine. Biofilms were washed with PBS and sonicated by probe sonicator (5 s on, 5 s off for 5 min in ice slurry, amplitude 30%) for cell lysis.

Protein samples were separated on a SDS–PAGE gel. Protein bands were washed with ddH_2_O mixed with 50% acetonitrile (ACN)/50% 25 mM NH_4_HCO_3_ via vigorous vortexing for 30 min, and dehydrated with 100% ACN until the gel particles became white. They were then reduced with 10 mM dithiothreitol at 56 °C for 1 h and alkylated with 55 mM iodoacetamide (IAA) for 45 min in the dark. The proteins were then washed with 25 mM NH_4_HCO_3_ and 50% ACN/50% 25 mM NH_4_HCO_3_. Gel particles were then dehydrated with 100% ACN and dried under vacuum. Trypsin (V5111, Promega, Madison, WI) was added to the gel particles at a ratio of 1:30, and allowed to be completely adsorbed by the gel particles. A unit of 25 mM NH_4_HCO_3_ was then added to completely cover the particles for incubation at 37 °C overnight. Peptides were extracted from the gel particles by two 20-min sonications in the presence of 50% ACN containing 0.1% Trifluoroacetic acid (TFA). Extracts were combined, vacuum-dried and resuspended in 0.1% FA for liquid chromatography (LC)–MS/MS analysis.

Peptides were separated and analysed on a Dionex Ultimate 3000 RSLCnano system coupled to a Q Exactive (Thermo Fisher, MA) as previously described^50^. Approximately 1 μg of peptide from each pooled fraction was injected into an Acclaim peptide trap column (Thermo Fisher, MA, USA) via the Dionex RSLCnano auto-sampler. Peptides were separated in a Dionex EASY-Spray 75 μm × 10 cm column packed with PepMap C18 3 μm, 100 Å (Thermo-Scientific, MA, USA) at room temperature. The flow rate was 300 nl min^−1^. Mobile phase A (0.1% formic acid in 5% ACN) and mobile phase B (0.1% formic acid in 90% ACN) were used to establish a 60-min gradient. Peptides were then analysed on Q Exactive with an EASY nanospray source (Thermo Fisher, MA) at an electrospray potential of 1.5 kV. A full MS scan (350–1,600 *m*/*z* range) was acquired at a resolution of 70,000 at *m*/*z* 200 and a maximum ion accumulation time of 100 ms. Dynamic exclusion was set as 15 s. The resolution of the higher energy collisional dissociation (HCD) spectra was set to 17,500 at *m*/*z* 200. The automatic gain control (AGC) settings of the full MS scan and the MS^2^ scan were 3E6 and 2E5, respectively. The 10 most intense ions above the 2,000 count threshold were selected for fragmentation in HCD, with a maximum ion accumulation time of 100 ms. An isolation width of 2 was used for MS^2^. Single and unassigned charged ions were excluded from MS/MS. For HCD, the normalized collision energy was set to 28%. The underfill ratio was defined as 0.2%.

Two injections have been performed for the first biological replicates and three injections have been performed for the second and third biological replicates, respectively, to evaluate the technical reproducibility of the instrument and workflow. Raw data files of the eight technical replicates were processed and searched as eight experiments using MaxQuant (v1.5.2.8) (refs 25, 26) and the Genebank *P. aeruginosa* protein database (downloaded on 21 May 2015, 55,063 sequences, 17,906,244 residues) together with the common contaminant proteins. Standard search type with 2 multiplicity, 3 maximum labelled AAs and heavy labelled Lys6 were used for the pulsed-SILAC quantitation. The database search was performed using the Andromeda search engine bundled with MaxQuant using the MaxQuant default parameters for Q Exactive Orbitrap mass spectrometer. The first and main searches peptide mass tolerance for were 20 and 4.5 parts per million (p.p.m.) respectively, while the MS/MS match tolerance was 20 p.p.m. with FTMS de-isotoping enabled. The absence of two trypsin cleavage sites per protein was allowed. Carbamidomethylation (C) was set as a fixed modification. Oxidation (M) and deamidation (NQ) were set as variable modifications. The search was performed in the Revert decoy mode with PSM FDR, protein FDR and site decoy fraction set to 0.01.

A total of 4,382 proteins including 4,250 *P. aeruginosa* proteins were identified by Andromeda in MaxQuant with FDR <1% (Supplementary Data 2). The scatter plots of inter-technical replicates of each biological replicate were used to evaluate the technical reproducibility of the results (Supplementary Data 2). The technical reproducibility is good and follows the order of BR2(*R*^2^=∼0.95)>BR3(*R*^2^=∼0.9)>BR1(*R*^2^=0.86). To evaluate the repeatability of the biological replicates, all LC–MS/MS raw data files of BR1, BR2 or BR3 were grouped and searched, respectively, using MaxQuant to obtain the pulsed-SILAC protein abundance levels in the three biological replicates. The scatter plots of inter-biological replicates were all included in Supplementary Data 2.

### Relative quantification of OdDHL

Effluents from flow chambers were collected, filtered through 0.2-μm filters and the filtrates were collected. Overnight cultures of the reporter strain Δ*lasI*Δ*rhlI*/*lasB-gfp* (ref. 51) were adjusted to OD_600_=0.15 using ABTGC medium. A volume of 100 μl of filtrate was added to 100 μl of Δ*lasI*Δ*rhlI*/*lasB-gfp* in a 96-well plate (Nunc, Denmark). Because Δ*lasI*Δ*rhlI* cannot produce its own OdDHL, *lasB-gfp* is induced by the addition of filtrates containing OdDHL. Fluorescence from *lasB-gfp* expression (expressed in r.f.u.) was measured for each well using a microplate reader (Tecan Infinite 2000) and was normalized to the OD_600_ of each well. Experiments were performed in triplicate, and results are shown as the mean±s.d.

### Elastase assay

Effluents from flow chambers were collected, centrifuged to remove bacterial cells and filtered through 0.2-μm filters. An Elastase Assay Kit (EnzChek) was used according to the manufacturer's instructions. ABTG medium was used as the negative control and elastase was used as the positive control. The fluorescence from each reaction (expressed in r.f.u.) was measured using a microplate reader (Tecan Infinite 2000) and was normalized to the OD_600_ for each well of the 96-well microplate. Experiments were performed in triplicate, and results are shown as the mean±s.d.

### Pyocyanin assay

Relative pyocyanin concentrations were quantified as described previously^52^. Effluents from flow chambers were collected, centrifuged to remove bacterial cells and filtered through 0.2-μm filters. ABTG medium was used as the negative control. A volume of 10 ml of filtrate was mixed with 1 ml of chloroform by vortexing. The lower chloroform phase was transferred to a new tube containing 200 μl of 0.2 M hydrochloric acid and vortexed. The top phase was then carefully transferred to a 96-well plate to measure OD_520_ using a microplate reader (Tecan Infinite 2000). Experiments were performed in triplicate, and the results are shown as the mean±s.d.

### Cytotoxicity assay for macrophages

In all, 5 × 10^5^ RAW264.7 (ATCC TIB-71) murine macrophages were grown in 24-well culture plate as previously described^38^. The effluent from the flow chamber biofilms with or without colistin treatment were collected and filtered through a 0.2-μm filter to remove bacterial cells. ABTG media was used as negative control, while ABTG media+10 μg ml^−1^ colistin was used to show that colistin had no side effects on macrophages. The resultant supernatant was mixed at equal volumes with fresh DMEM and added to the macrophages. Macrophages were incubated for 4 h in 37 °C, 5% CO_2_. The cytotoxicity of the supernatants on macrophages was determined by adding 20 μM PI to monitor cell integrity. Macrophages stained red by PI under epifluorescent microscopy ( × 20 objective) were counted as dead, leaving the live ones unstained. Cells from five images of each samples were enumerated and the ratio of dead cells to live cells was calculated. Experiments were performed in triplicate, and the results are shown as the mean±s.d.

### Eradication of biofilms with colistin and DIPY iron chelator

Fluorescent-tagged *P. aeruginosa* biofilms were cultivated as described above. After 72 h of growth, PAO1 biofilms were treated with 10 μg ml^−1^ colistin or 100 μg ml^−1^ DIPY plus 10 μg ml^−1^ colistin. After 48 h of treatment, 300 μl of 20 μM PI was injected into each flow channel to stain dead cells in the biofilm. Biofilm were observed under the CLSM as described above. As pyoverdine is a fluorescent metabolite^53^, its presence and localization in the biofilm was observed by CLSM with Ex 358 nm/Em 461 nm. Experiments were performed in triplicate, and results are shown as the mean±s.d.

### Eradication of biofilms by colistin and erythromycin

Fluorescent-tagged *P. aeruginosa* biofilms were cultivated as described above. After 72 h of growth, PAO1 biofilms were treated with 10 μg ml^−1^ colistin or 100 μg ml^−1^ erythromycin plus 10 μg ml^−1^ colistin. After 48 h of treatment, 300 μl of 20 μM PI was injected into each flow channel to stain dead cells in the biofilm. Biofilm were observed under the CLSM as described above. Experiments were performed in triplicate, and results are shown as the mean±s.d.

### Time-dependent killing of planktonic PAO1 with erythromycin and colistin

The minimal inhibitory concentration (MIC) of erythromycin is 2,000 μg ml^−1^, while the MIC for colistin is 1 μg ml^−1^ in PAO1 strains. Planktonic *P. aeruginosa* strains were grown at 37 °C in ABTGC with 100 μg ml^−1^ of erythromycin, 10 μg ml^−1^ of colistin and 100 μg ml^−1^ of erythromycin+10 μg ml^−1^ of colistin. Cells were collected at 0, 1 and 3 h. PAO1 cells were serially diluted, plated on LB agar and incubated at 37 °C overnight. C.f.u. per ml was calculated by multiplying the average number of colonies by the dilution factor and dividing by the volume.

### Mouse implant infection model

The animals used in this study were 7-week-old female BALB/c mice (Taconic M&B A/S). The animal experiments were performed in accordance to the NACLAR Guidelines and Animal and Birds (Care and Use of Animals for Scientific Purposes) Rules by Agri-Food & Authority of Singapore (AVA), with authorization and approval by the Institutional Care and Use Committee (IACUC) and Nanyang Technological University, under the permit number A-0191 AZ. PAO1 biofilms were grown on cylindrical implants (3 mm × 5 mm Ø) in 0.9% NaCl at 37 °C with shaking at 110 r.p.m. for 20 h, as described previously^40^,^54^. The biofilm-coated implants were washed three times with 0.9% NaCl and transplanted into the peritoneum of the mice after they were anesthetized with 100 mg kg^−1^ ketamine and 10 mg kg^−1^ xylene. Antibiotics were injected at the site of implantation. No antibiotics, 1 mg kg^−1^ colistin, 10 mg kg^−1^ erythromycin, or 1 mg kg^−1^ colistin and 10 mg kg^−1^ erythromycin were used on groups of five mice. Mice were killed after 24 h of treatment. Implants were collected, sonicated in 0.9% NaCl in an ice water bath using an Elmasonic P120H (Elma, Germany; power=50% and frequency=37 KHz) for 10 min and vortexed three times for 10 s to disrupt the biofilms. Spleens were also collected and completely homogenized using the Bio-Gen PRO200 Homogenizer (Pro Scientific, US) at maximum power on ice. Samples were serially diluted, plated on LB agar and incubated at 37 °C overnight. C.f.u. per ml was calculated by multiplying the average number of colonies by the dilution factor and multiplying by the volume. Experiments were performed with five replicates, and the results are shown as the mean±s.d.

## Additional information

**Accession codes:** Whole-genome sequence data for *P. aeruginosa* biofilms have been deposited in the NCBI Short Read Archive (SRA) database with accession code SRP058610. LC–MS/MS raw data of the eight replicates and results for protein and peptide identification and quantification from MaxQuant have been submitted to the ProteomeXchange Consortium via the PRIDE data repository with the dataset identifier PXD002369.

**How to cite this article:** Chua, S. L. *et al.* Selective labelling and eradication of antibiotic-tolerant bacterial populations in *Pseudomonas aeruginosa* biofilms. *Nat. Commun.* 7:10750 doi: 10.1038/ncomms10750 (2016).

## Supplementary Material

Supplementary Information Supplementary Figures 1-5, Supplementary Table 1, Supplementary Methods and Supplementary References

Supplementary Movie 1 Killing of biofilms by colistin. Top view of 72 h old wild-type *P. aeruginosa* biofilms were treated with colistin and observed from 2 to 20 h. Scale bars, 10 μm.

Supplementary Movie 2 Killing of biofilms by colistin. Side view of 72 h old wild-type *P. aeruginosa* biofilms were treated with colistin and observed from 2 to 20 h. Scale bars, 10 μm.

Supplementary Movie 3 Migration and formation of colistin tolerant subpopulation in biofilm. Top view of 72 h old wild-type *P. aeruginosa* biofilms were treated with colistin and observed from 24 to 34 h of treatment. Scale bars, 10 μm.

Supplementary Movie 4 Migration and formation of colistin tolerant subpopulation in biofilm. Side view of 72 h old wild-type *P. aeruginosa* biofilms were treated with colistin and observed from 24 to 34 h of treatment. Scale bars, 10 μm.

Supplementary Data 1 Comparing the variants of colistin-treated biofilms samples with untreated biofilm samples.

Supplementary Data 2 Total list of *P. aeruginosa* proteins identifed by SILAC.

Supplementary Data 3 Proteins that were of lower abundance in *P. aeruginosa* PAO1 tolerant cells than in non-tolerant biofilm cells.

Supplementary Data 4 Proteins that were of higher abundance in *P. aeruginosa* PAO1 tolerant cells than in non-tolerant biofilm cells.

## References

[b1] Neu H. C. The crisis in antibiotic resistance. Science 257, 1064–1073 (1992). 1509257 10.1126/science.257.5073.1064

[b2] Lewis K. Persister cells, dormancy and infectious disease. Nat. Rev. Microbiol. 5, 48–56 (2007). 17143318 10.1038/nrmicro1557

[b3] Balaban N. Q., Merrin J., Chait R., Kowalik L. & Leibler S. Bacterial persistence as a phenotypic switch. Science 305, 1622–1625 (2004). 15308767 10.1126/science.1099390

[b4] Harrison J. J., Turner R. J. & Ceri H. Persister cells, the biofilm matrix and tolerance to metal cations in biofilm and planktonic Pseudomonas aeruginosa. Environ. Microbiol. 7, 981–994 (2005). 15946294 10.1111/j.1462-2920.2005.00777.x

[b5] Spoering A. L. & Lewis K. Biofilms and planktonic cells of Pseudomonas aeruginosa have similar resistance to killing by antimicrobials. J. Bacteriol. 183, 6746–6751 (2001). 11698361 10.1128/JB.183.23.6746-6751.2001 PMC95513

[b6] Costerton J. W., Lewandowski Z., Caldwell D. E., Korber D. R. & Lappin-Scott H. M. Microbial biofilms. Annu. Rev. Microbiol. 49, 711–745 (1995). 8561477 10.1146/annurev.mi.49.100195.003431

[b7] Leid J. G. *et al.* The exopolysaccharide alginate protects Pseudomonas aeruginosa biofilm bacteria from IFN-gamma-mediated macrophage killing. J. Immunol. 175, 7512–7518 (2005). 16301659 10.4049/jimmunol.175.11.7512

[b8] Colvin K. M. *et al.* The pel polysaccharide can serve a structural and protective role in the biofilm matrix of Pseudomonas aeruginosa. PLoS Pathog. 7, e1001264 (2011). 21298031 10.1371/journal.ppat.1001264 PMC3029257

[b9] Chua S. L. *et al.* C-di-GMP regulates Pseudomonas aeruginosa stress response to tellurite during both planktonic and biofilm modes of growth. Sci. Rep. 5, 10052 (2015). 25992876 10.1038/srep10052 PMC4438720

[b10] Parsek M. R. & Tolker-Nielsen T. Pattern formation in Pseudomonas aeruginosa biofilms. Curr. Opin. Microbiol. 11, 560–566 (2008). 18935979 10.1016/j.mib.2008.09.015

[b11] Dawson C. C., Intapa C. & Jabra-Rizk M. A. "Persisters": survival at the cellular level. PLoS Pathog. 7, e1002121 (2011). 21829345 10.1371/journal.ppat.1002121 PMC3145784

[b12] Allison K. R., Brynildsen M. P. & Collins J. J. Metabolite-enabled eradication of bacterial persisters by aminoglycosides. Nature 473, 216–220 (2011). 21562562 10.1038/nature10069 PMC3145328

[b13] Conlon B. P. *et al.* Activated ClpP kills persisters and eradicates a chronic biofilm infection. Nature 503, 365–370 (2013). 24226776 10.1038/nature12790 PMC4031760

[b14] Vega N. M., Allison K. R., Khalil A. S. & Collins J. J. Signaling-mediated bacterial persister formation. Nat. Chem. Biol. 8, 431–433 (2012). 22426114 10.1038/nchembio.915 PMC3329571

[b15] Shah D. *et al.* Persisters: a distinct physiological state of *E. coli*. BMC Microbiol. 6, 53 (2006). 16768798 10.1186/1471-2180-6-53 PMC1557402

[b16] Talbot G. H. *et al.* Bad bugs need drugs: an update on the development pipeline from the Antimicrobial Availability Task Force of the Infectious Diseases Society of America. Clin. Infect. Dis. 42, 657–668 (2006). 16447111 10.1086/499819

[b17] Li J. *et al.* Colistin: the re-emerging antibiotic for multidrug-resistant Gram-negative bacterial infections. Lancet Infect. Dis. 6, 589–601 (2006). 16931410 10.1016/S1473-3099(06)70580-1

[b18] Haagensen J. A. *et al.* Differentiation and distribution of colistin- and sodium dodecyl sulfate-tolerant cells in Pseudomonas aeruginosa biofilms. J. Bacteriol. 189, 28–37 (2007). 17041046 10.1128/JB.00720-06 PMC1797205

[b19] Walters M. C. 3rd, Roe F., Bugnicourt A., Franklin M. J. & Stewart P. S. Contributions of antibiotic penetration, oxygen limitation, and low metabolic activity to tolerance of Pseudomonas aeruginosa biofilms to ciprofloxacin and tobramycin. Antimicrob. Agents Chemother. 47, 317–323 (2003). 12499208 10.1128/AAC.47.1.317-323.2003 PMC148957

[b20] Stewart P. S. Mechanisms of antibiotic resistance in bacterial biofilms. Int. J. Med. Microbiol. 292, 107–113 (2002). 12195733 10.1078/1438-4221-00196

[b21] Hengzhuang W., Wu H., Ciofu O., Song Z. & Hoiby N. Pharmacokinetics/pharmacodynamics of colistin and imipenem on mucoid and nonmucoid Pseudomonas aeruginosa biofilms. Antimicrob. Agents Chemother. 55, 4469–4474 (2011). 21670181 10.1128/AAC.00126-11 PMC3165294

[b22] Ong S. E. *et al.* Stable isotope labeling by amino acids in cell culture, SILAC, as a simple and accurate approach to expression proteomics. Mol. Cell. Proteomics 1, 376–386 (2002). 12118079 10.1074/mcp.m200025-mcp200

[b23] Phillips N. J. *et al.* Proteomic analysis of Neisseria gonorrhoeae biofilms shows shift to anaerobic respiration and changes in nutrient transport and outermembrane proteins. PloS ONE 7, e38303 (2012). 22701624 10.1371/journal.pone.0038303 PMC3368942

[b24] Post D. *et al.* Comparative analyses of proteins from Haemophilus influenzae biofilm and planktonic populations using metabolic labeling and mass spectrometry. BMC Microbiol. 14, 2322 (2014). 10.1186/s12866-014-0329-9 PMC4302520 25551439

[b25] Cox J. *et al.* Andromeda: a peptide search engine integrated into the MaxQuant environment. J. Proteome Res. 10, 1794–1805 (2011). 21254760 10.1021/pr101065j

[b26] Tyanova S., Mann M. & Cox J. MaxQuant for in-depth analysis of large SILAC datasets. Methods Mol. Biol. 1188, 351–364 (2014). 25059623 10.1007/978-1-4939-1142-4_24

[b27] Pamp S. J., Gjermansen M., Johansen H. K. & Tolker-Nielsen T. Tolerance to the antimicrobial peptide colistin in Pseudomonas aeruginosa biofilms is linked to metabolically active cells, and depends on the pmr and mexAB-oprM genes. Mol. Microbiol. 68, 223–240 (2008). 18312276 10.1111/j.1365-2958.2008.06152.x

[b28] Fernandez L. *et al.* Adaptive resistance to the ‘last hope' antibiotics polymyxin B and colistin in Pseudomonas aeruginosa is mediated by the novel two-component regulatory system ParR-ParS. Antimicrob. Agents Chemother. 54, 3372–3382 (2010). 20547815 10.1128/AAC.00242-10 PMC2916309

[b29] Koo J. *et al.* PilF is an outer membrane lipoprotein required for multimerization and localization of the Pseudomonas aeruginosa Type IV pilus secretin. J. Bacteriol. 190, 6961–6969 (2008). 18776008 10.1128/JB.00996-08 PMC2580691

[b30] Parsek M. R. & Greenberg E. P. Sociomicrobiology: the connections between quorum sensing and biofilms. Trends Microbiol. 13, 27–33 (2005). 15639629 10.1016/j.tim.2004.11.007

[b31] Hentzer M. *et al.* Inhibition of quorum sensing in Pseudomonas aeruginosa biofilm bacteria by a halogenated furanone compound. Microbiology 148, 87–102 (2002). 11782502 10.1099/00221287-148-1-87

[b32] Jensen P. O. *et al.* Rapid necrotic killing of polymorphonuclear leukocytes is caused by quorum-sensing-controlled production of rhamnolipid by Pseudomonas aeruginosa. Microbiology 153, 1329–1338 (2007). 17464047 10.1099/mic.0.2006/003863-0

[b33] Alhede M. *et al.* Pseudomonas aeruginosa recognizes and responds aggressively to the presence of polymorphonuclear leukocytes. Microbiology 155, 3500–3508 (2009). 19643762 10.1099/mic.0.031443-0

[b34] Van Gennip M. *et al.* Inactivation of the rhlA gene in Pseudomonas aeruginosa prevents rhamnolipid production, disabling the protection against polymorphonuclear leukocytes. APMIS 117, 537–546 (2009). 19594494 10.1111/j.1600-0463.2009.02466.x PMC2997331

[b35] Lau G. W., Hassett D. J., Ran H. & Kong F. The role of pyocyanin in Pseudomonas aeruginosa infection. Trends Mol. Med. 10, 599–606 (2004). 15567330 10.1016/j.molmed.2004.10.002

[b36] Nomura K. *et al.* Pseudomonas aeruginosa elastase causes transient disruption of tight junctions and downregulation of PAR-2 in human nasal epithelial cells. Respir. Res. 15, 21 (2014). 24548792 10.1186/1465-9921-15-21 PMC3936699

[b37] Yang L., Nilsson M., Gjermansen M., Givskov M. & Tolker-Nielsen T. Pyoverdine and PQS mediated subpopulation interactions involved in Pseudomonas aeruginosa biofilm formation. Mol. Microbiol. 74, 1380–1392 (2009). 19889094 10.1111/j.1365-2958.2009.06934.x

[b38] Chua S. L. *et al.* Dispersed cells represent a distinct stage in the transition from bacterial biofilm to planktonic lifestyles. Nat. Commun. 5, 4462 (2014). 25042103 10.1038/ncomms5462

[b39] Perez-Martinez I. & Haas D. Azithromycin inhibits expression of the GacA-dependent small RNAs RsmY and RsmZ in Pseudomonas aeruginosa. Antimicrob. Agents Chemother. 55, 3399–3405 (2011). 21537014 10.1128/AAC.01801-10 PMC3122407

[b40] Christensen L. D. *et al.* Impact of Pseudomonas aeruginosa quorum sensing on biofilm persistence in an in vivo intraperitoneal foreign-body infection model. Microbiology 153, 2312–2320 (2007). 17600075 10.1099/mic.0.2007/006122-0

[b41] Schierholz J. M. & Beuth J. Implant infections: a haven for opportunistic bacteria. J. Hosp. Infect. 49, 87–93 (2001). 11567552 10.1053/jhin.2001.1052

[b42] Costerton J. W., Montanaro L. & Arciola C. R. Biofilm in implant infections: its production and regulation. Int. J. Artif. Organs 28, 1062–1068 (2005). 16353112 10.1177/039139880502801103

[b43] Bjarnsholt T. *et al.* Pseudomonas aeruginosa biofilms in the respiratory tract of cystic fibrosis patients. Pediatr. Pulmonol. 44, 547–558 (2009). 19418571 10.1002/ppul.21011

[b44] Bjarnsholt T. *et al.* The *in vivo* biofilm. Trends Microbiol. 21, 466–474 (2013). 23827084 10.1016/j.tim.2013.06.002

[b45] Moreau-Marquis S. *et al.* The DeltaF508-CFTR mutation results in increased biofilm formation by Pseudomonas aeruginosa by increasing iron availability. Am. J. Physiol. Lung Cell. Mol. Physiol. 295, L25–L37 (2008). 18359885 10.1152/ajplung.00391.2007 PMC2494796

[b46] Moreau-Marquis S., O'Toole G. A. & Stanton B. A. Tobramycin and FDA-approved iron chelators eliminate Pseudomonas aeruginosa biofilms on cystic fibrosis cells. Am. J. Respir. Cell. Mol. Biol. 41, 305–313 (2009). 19168700 10.1165/rcmb.2008-0299OC PMC2742750

[b47] Wu A. *et al.* Cell motility and drug gradients in the emergence of resistance to chemotherapy. Proc. Natl Acad. Sci. USA 110, 16103–16108 (2013). 24046372 10.1073/pnas.1314385110 PMC3791735

[b48] Chua S. L. *et al.* Bis-(3′-5′)-cyclic dimeric GMP regulates antimicrobial peptide resistance in Pseudomonas aeruginosa. Antimicrob. Agents Chemother. 57, 2066–2075 (2013). 23403434 10.1128/AAC.02499-12 PMC3632963

[b49] Sternberg C. & Tolker-Nielsen T. Growing and analyzing biofilms in flow cells. Curr. Protoc. Microbiol. Chapter 1, Unit 1B 2 (2006). 10.1002/9780471729259.mc01b02s00 18770573

[b50] Hao P. *et al.* Deep proteomic profiling of human carotid atherosclerotic plaques using multidimensional LC-MS/MS. Proteomics Clin. Appl. 8, 631–635 (2014). 24828403 10.1002/prca.201400007

[b51] Hansen S. K. *et al.* Evolution and diversification of Pseudomonas aeruginosa in the paranasal sinuses of cystic fibrosis children have implications for chronic lung infection. ISME J. 6, 31–45 (2012). 21716309 10.1038/ismej.2011.83 PMC3246239

[b52] Mavrodi D. V. *et al.* Functional analysis of genes for biosynthesis of pyocyanin and phenazine-1-carboxamide from Pseudomonas aeruginosa PAO1. J. Bacteriol. 183, 6454–6465 (2001). 11591691 10.1128/JB.183.21.6454-6465.2001 PMC100142

[b53] Elliott R. P. Some properties of pyoverdine, the water-soluble fluorescent pigment of the pseudomonads. Appl. Microbiol. 6, 241–246 (1958). 13559972 10.1128/am.6.4.241-246.1958 PMC1057401

[b54] Chua S. L. *et al.* *In vitro* and *in vivo* generation and characterization of Pseudomonas aeruginosa biofilm-dispersed cells via c-di-GMP manipulation. Nat. Protoc. 10, 1165–1180 (2015). 26158442 10.1038/nprot.2015.067

