# Supplementary material for: Selective labelling and eradication of antibiotic-tolerant bacterial populations in Pseudomonas aeruginosa biofilms
Source: Nat Commun. 2016 Feb 19;7:10750. doi: 10.1038/ncomms10750 (PMC4762895; doi:10.1038/ncomms10750)
Supplement: Supplementary Information — Supplementary Figures 1-5, Supplementary Table 1, Supplementary Methods and Supplementary References [file ncomms10750-s1.pdf]

Supplementary Figures

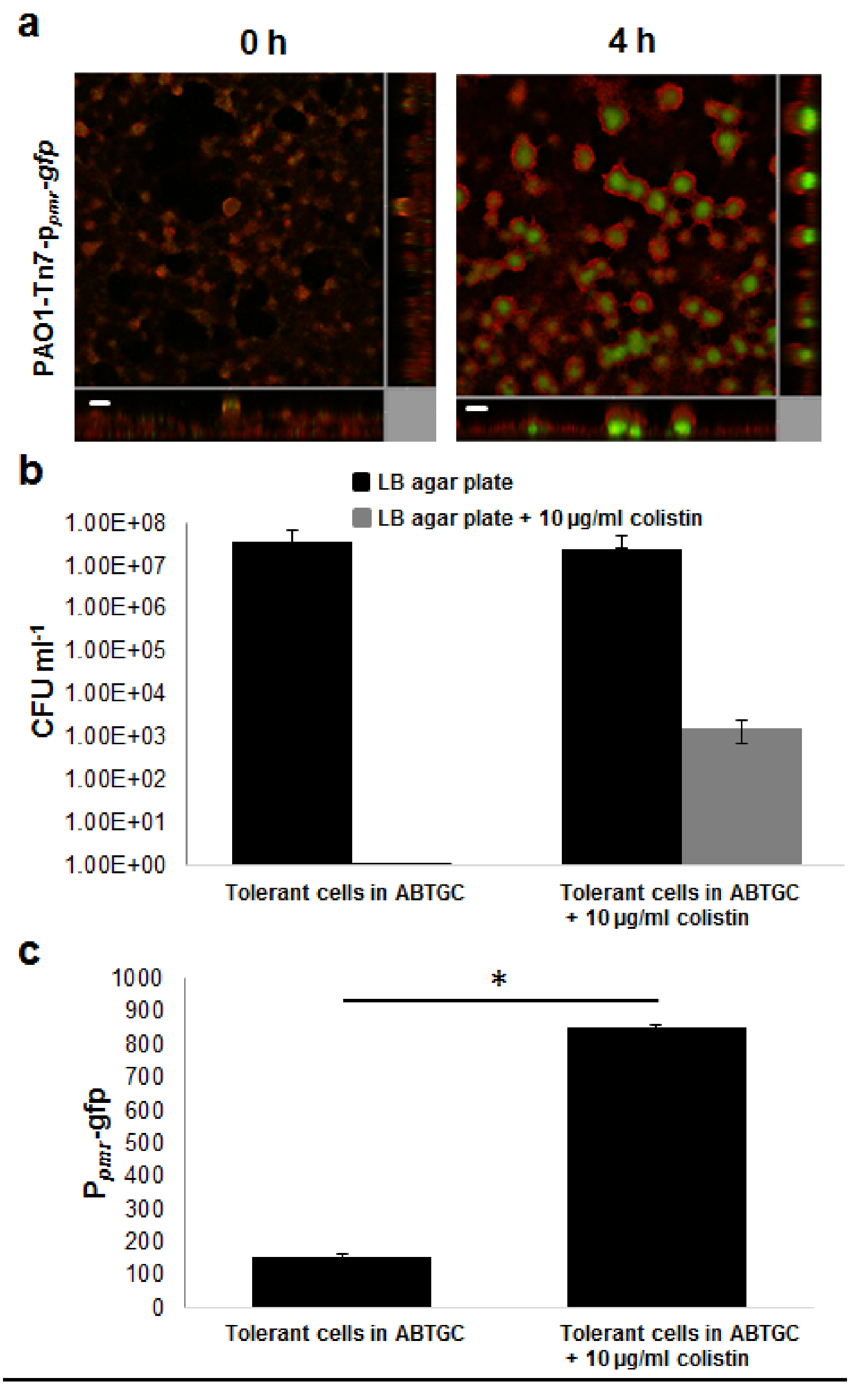

Supplementary Figure 1. Colistin-tolerant phenotype of the antibiotic tolerant biofilm cells is reversible. (a) Expression of GFP in 72 h-old Tn7-P$_{pmr}$-*gfp* - tagged PAO1 biofilms treated by ABTG containing 10 µg mL^{-1} colistin for 4 h. Total biomass of biofilms was stained red by CYTO62 and cells had induced P$_{pmr}$-*gfp*

expression appeared green. Experiments were performed in triplicate, and a representative image for each condition is shown. Scale bars, 10 µm. (b) Colistin-tolerant cells obtained from the biofilm were homogenized and cultivated in ABTGC medium with and without 10 µg ml^{-1} colistin and plated on LB agar plates with and without 10 µg ml^{-1} colistin. CFU ml^{-1} was calculated after incubation. (c) Expression of p_{pmr}-*gfp* by colistin-tolerant cells obtained from the biofilm after homogenization and cultivation in ABTGC medium with and without 10 µg ml^{-1} colistin. The mean and s.d. from three experiments is shown. *$P < 0.01$, Student's t-test.

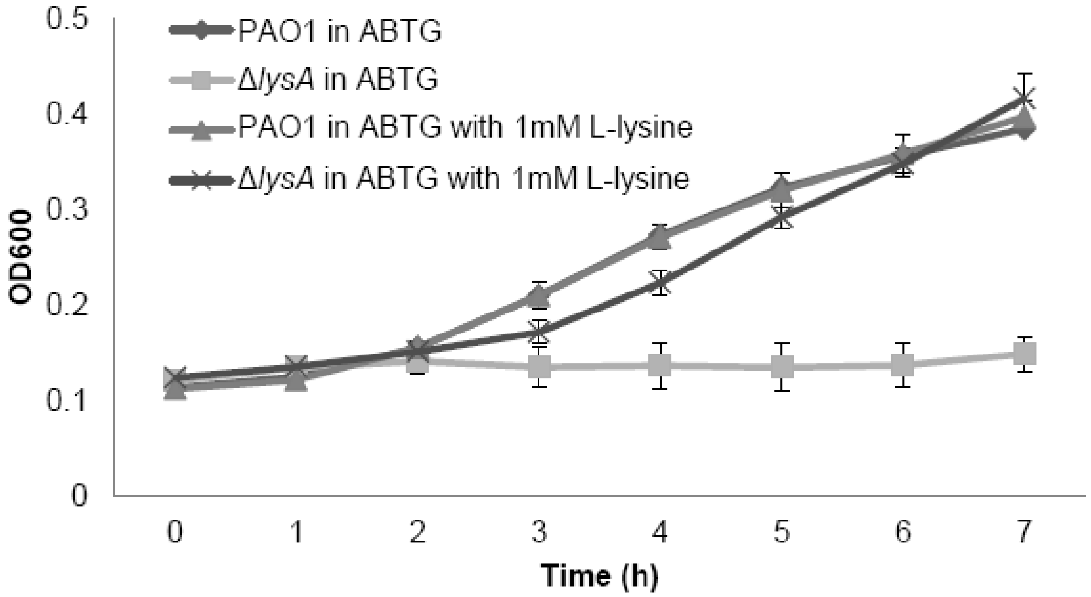

Supplementary Figure 2. Growth curves of PAO1 and Δ*lysA* mutant. Δ*lysA* mutant did not grow in ABTG medium lacking L-lysine. Supplementing 1 mM L-lysine to ABTG medium allowed Δ*lysA* to grow at the same rate as PAO1.

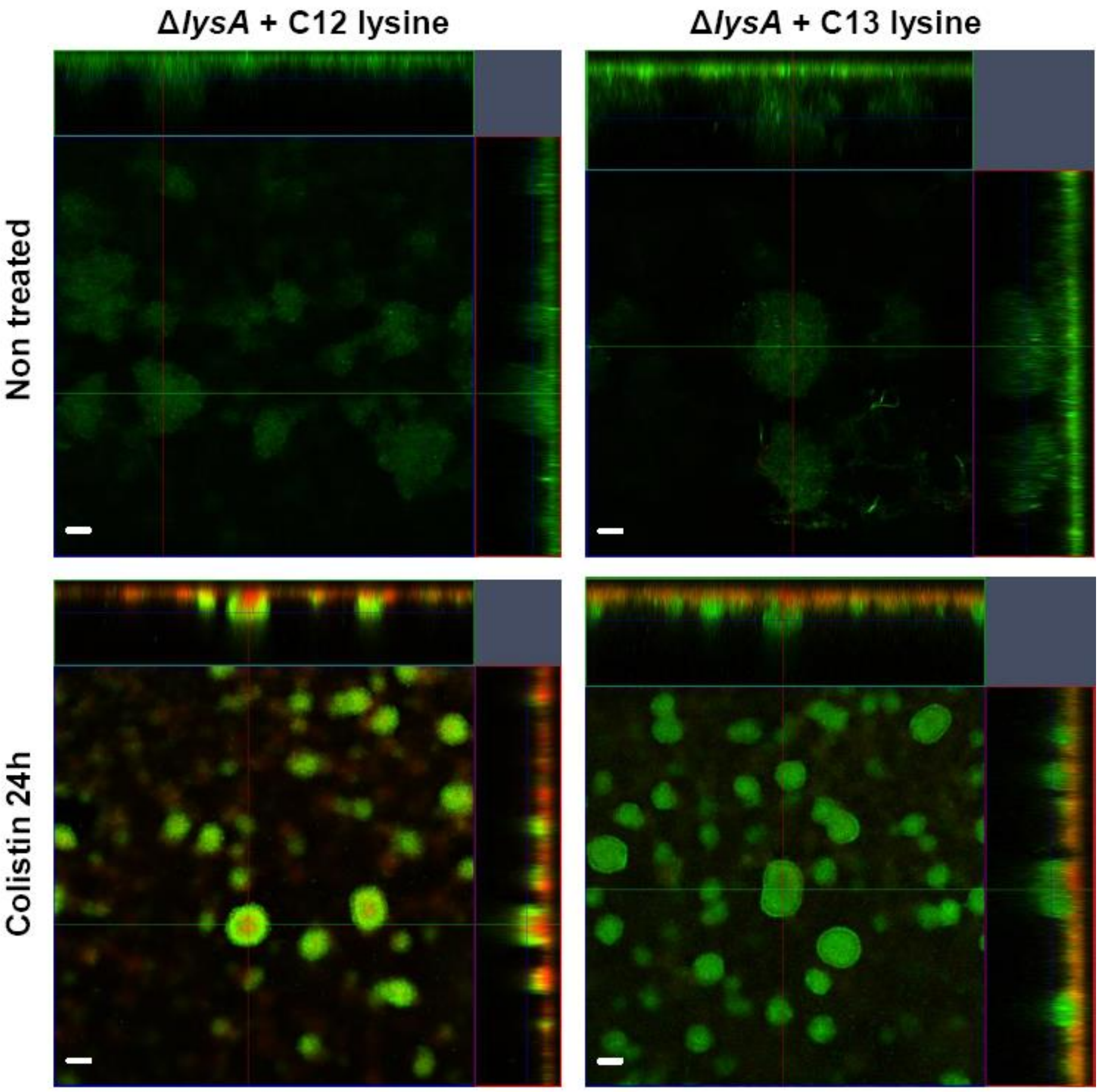

Supplementary Figure 3. Comparison of Δ*lysA* biofilm formation and antibiotic tolerant cell development in media containing C12 or C13 L-lysine. Biofilms were grown in ABTG media containing C12 or C13 L-lysine for 72 h. There was no difference in the development of antibiotic tolerant cells in biofilms cultivated in media containing C12 and C13 L-lysine. Experiments were performed in triplicate, and a representative image for each condition is shown. Live cells appear green, whereas dead cells appear yellow or red.

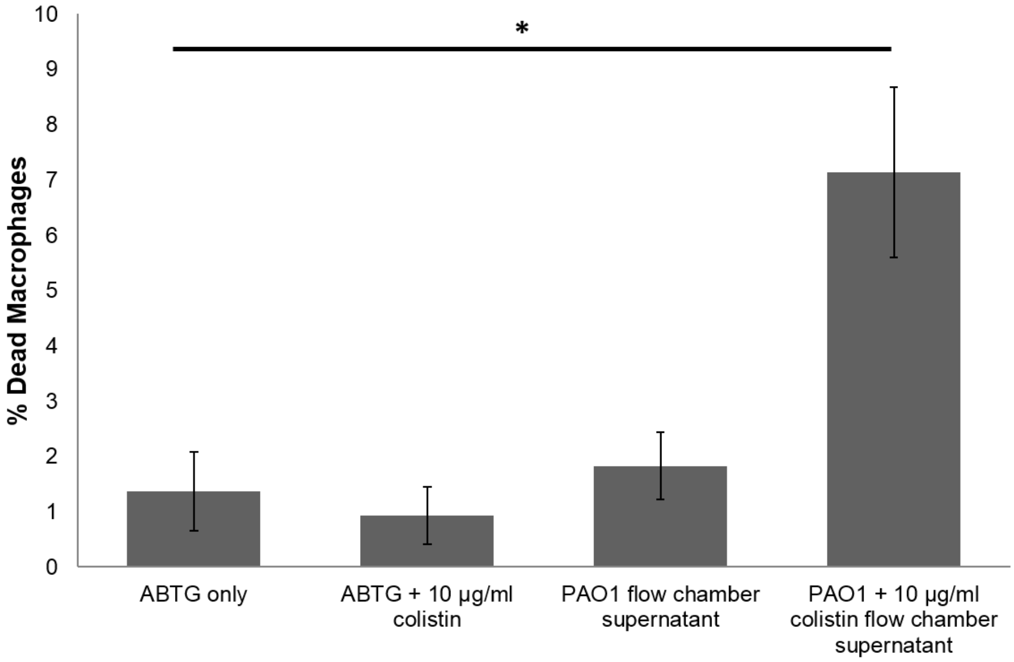

Supplementary Figure 4. Supernatant from colistin-tolerant flow chamber biofilms are cytotoxic to macrophages. The macrophages were treated with ABTG medium as control and compared with supernatants from PAO1 flow chamber biofilms treated with or without 10 µg ml^{-1} colistin. ABTG + 10 µg ml^{-1} colistin was also tested to observe if colistin itself has any cytotoxicity effect on macrophages. The mean and s.d. from three experiments is shown. *$P < 0.01$, Student's t-test.

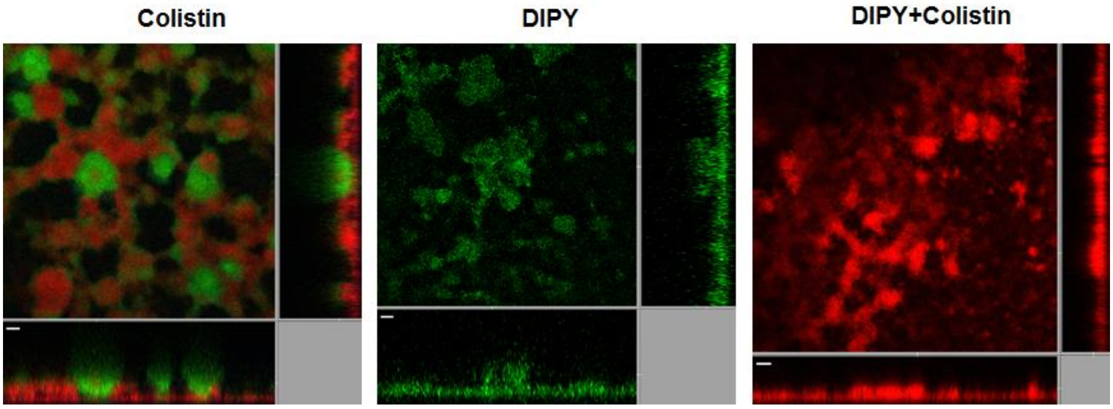

Supplementary Figure 5. DIPY interferes migration of colistin-tolerant cell aggregates to the top of dead biofilms. Biofilms were cultivated for 72 h using *P. aeruginosa* PAO1, followed by treatment with medium containing 10 µg ml^{-1} colistin, 100 µg mL^{-1} DIPY or 100 µg mL^{-1} DIPY + 10 µg ml^{-1} colistin. No migration of tolerant subpopulation was observed for biofilms treated by 100 µg mL^{-1} DIPY + 10 µg ml^{-1} colistin. The central images show horizontal optical sections, whereas the flanking images show vertical optical sections. Live cells appear green and dead cells appear red. Scale Bars, 10 µm.

Supplementary Table 1.

**Strains**	Relevant characteristic(s)	Source or reference [a]
***P. aeruginosa* strains**		
PAO1	Prototypic nonmucoid wild-type strain	[1]
PAO1[a]	Prototypic nonmucoid wild-type strain	[2]
Δ*lysA*	Tc^r; L-lysine synthesis defective *lysA* mutant in PAO1[a]	[2]
PAO1/*pmr-gfp*	Gm^r; PAO1 containing the *pmr-gfp* translational fusion	[3]
PAO1/*lasB-gfp*	Gm^r; PAO1 containing the *lasB-gfp* translational fusion	[4]
Δ*pilA*	Tel^r; *pilA* mutant of PAO1	[5]
Δ*lasI*Δ*rhlI*	Gm^r; Tc^r; *lasI* and *rhlI* double mutant of PAO1	[6]
Δ*lasI*Δ*rhlI*/*lasB-gfp*	Gm^r; Tc^r; *lasI* and *rhlI* double mutant of PAO1 containing the *lasB-gfp* translational fusion	[7]
Δ*pilA*Δ*lasR*Δ*rhlR*	Tel^r; Gm^r; Tc^r; *pilA*, *lasI* and *rhlI* double mutant of PAO1	[5]
Δ*pilA*/pDA2	Tel^r; $Carb^r$; *pilA* mutant of PAO1 carrying the pDA2 plasmid (pUCP22/*pilA*)	[8]

Bacterial strains used in this study.

Supplementary Methods

Parameters used in the CLC genomics analysis

1. Import reads using Illumina pared importer:

    Discard read names = Yes

    Discard quality scores = No

    Paired orientation = Paired reads (forward-reverse)

    Minimum distance = 1

    Maximum distance = 500

    Quality score = NCBI/Sanger or Illumina Pipeline 1.8 and later

    Remove failed reads = Yes

    MiSeq de-multiplexing = No

2. Trim sequences

Trim adapter list = Illumina TruSeq Trim Adapter List

    Ambiguous trim = Yes

    Ambiguous limit = 2

    Quality trim = Yes

    Quality limit = 0.05

    Use colorspace = No

    Create report = Yes

    Also search on reversed sequence = Yes

    Save discarded sequences = Yes

    Remove 5' terminal nucleotides = No

Minimum number of nucleotides in reads = 30

Discard short reads = Yes

Remove 3' terminal nucleotides = No

Discard long reads = No

Save broken pairs = No

3. Map reds to reference:

References = NC_002516(PA01 genome)

Masking mode = No masking

Mismatch cost = 2

Cost of insertions and deletions = Linear gap cost

Insertion cost = 3

Deletion cost = 3

Insertion open cost = 6

Insertion extend cost = 1

Deletion open cost = 6

Deletion extend cost = 1

Length fraction = 0.5

Similarity fraction = 0.8

Global alignment = No

Auto-detect paired distances = Yes

Non-specific match handling = Map randomly

Output mode = Create reads track

Create report = Yes

Collect un-mapped reads = No

4. Low frequency variant detection:

   Required significance (%) = 1.0

   Ignore positions with coverage above = 100,000

   Restrict calling to target regions = Not set

   Ignore broken pairs = Yes

   Ignore non-specific matches = Reads

   Minimum coverage = 10

   Minimum count = 2

   Minimum frequency (%) = 1.0

   Base quality filter = Yes

   Neighborhood radius = 5

   Minimum central quality = 20

   Minimum neighborhood quality = 15

   Read direction filter = Yes

   Direction frequency (%) = 5.0

   Relative read direction filter = Yes

   Significance (%) = 1.0

   Read position filter = Yes

   Significance (%) = 1.0

   Remove pyro-error variants = Yes

   In homopolymer regions with minimum length = 3

   With frequency below = 0.8

Create track = Yes

Create annotated table = Yes

Create report = Yes

Supplementary References:

1. Holloway BW, Morgan AF. Genome organization in Pseudomonas. *Annual review of microbiology* **40**, 79-105 (1986).

2. Jacobs MA, *et al.* Comprehensive transposon mutant library of Pseudomonas aeruginosa. *Proc Natl Acad Sci U S A* **100**, 14339-14344 (2003).

3. Haagensen JA, *et al.* Differentiation and distribution of colistin- and sodium dodecyl sulfate-tolerant cells in Pseudomonas aeruginosa biofilms. *J Bacteriol* **189**, 28-37 (2007).

4. Hentzer M, *et al.* Inhibition of quorum sensing in Pseudomonas aeruginosa biofilm bacteria by a halogenated furanone compound. *Microbiology* **148**, 87-102 (2002).

5. Barken KB, *et al.* Roles of type IV pili, flagellum-mediated motility and extracellular DNA in the formation of mature multicellular structures in Pseudomonas aeruginosa biofilms. *Environ Microbiol* **10**, 2331-2343 (2008).

6. Allesen-Holm M, *et al.* A characterization of DNA release in Pseudomonas aeruginosa cultures and biofilms. *Mol Microbiol* **59**, 1114-1128 (2006).

7. Hansen SK, *et al.* Evolution and diversification of Pseudomonas aeruginosa in the paranasal sinuses of cystic fibrosis children have implications for chronic lung infection. *The ISME journal* **6**, 31-45 (2012).

8. Yang L, Liu Y, Markussen T, Hoiby N, Tolker-Nielsen T, Molin S. Pattern differentiation in co-culture biofilms formed by Staphylococcus aureus and Pseudomonas aeruginosa. *FEMS Immunol Med Microbiol* **62**, 339-347 (2011).
